# The Predictive Value of Serum Squamous Cell Carcinoma Antigen in Patients with Cervical Cancer Who Receive Neoadjuvant Chemotherapy followed by Radical Surgery: A Single-Institute Study

**DOI:** 10.1371/journal.pone.0122361

**Published:** 2015-04-10

**Authors:** Xiong Li, Jin Zhou, Kecheng Huang, Fangxu Tang, Hang Zhou, Shaoshuai Wang, Yao Jia, Haiying Sun, Ding Ma, Shuang Li

**Affiliations:** 1 Department of Obstetrics and Gynecology, Tongji Hospital, Tongji Medical College, Huazhong University of Science and Technology, Wuhan, P. R. China; 2 Cancer Center, Renmin Hospital of Wuhan University, Wuhan, Hubei, P. R. China; Rajiv Gandhi Centre for Biotechnology, INDIA

## Abstract

**Objective:**

Neoadjuvant chemotherapy (NACT) could affect the levels of squamous cell carcinoma antigen (SCC-Ag). This study evaluates the predictive value of pre- and posttreatment SCC-Ag levels in patients with cervical cancer who were treated with NACT followed by radical surgery.

**Methods:**

A total of 286 patients with Stage IB1-IIIB squamous cell carcinoma of the uterine cervix who were treated with NACT followed by radical hysterectomy were analyzed retrospectively. The relationship between SCC-Ag levels, the clinicopathologic parameters, the response to NACT and the three-year survival rate was investigated.

**Results:**

The levels of SCC-Ag were elevated (>3.5 ng/mL) in 43.8% of patients before NACT, and 13.0% of patients after NACT. Pre- and posttreatment levels of SCC-Ag correlated with the response to NACT (*P* = 0.010, and *P*<0.001), deep stromal infiltration (*P* = 0.041, and *P* = 0.006), and lymph node status (*P*<0.001, and *P*<0.001). In the multivariate analysis, the elevated pretreatment level of SCC-Ag was demonstrated to be an independent risk factor for Lymph node metastases (*P*<0.001). Patients with both pre- and posttreatment SCC-Ag levels ≤3.5 ng/mL showed the best 3-year disease-free survival (DFS) and 3-year overall survival (OS) compared with patients with either pre- or posttreatment levels >3.5 ng/mL (*P*<0.001, and *P*<0.001, respectively). A multivariate analysis showed that posttreatment SCC-Ag levels were a strong independent predictor of OS (*P* = 0.001) and DFS (*P* = 0.012).

**Conclusion:**

Elevated pretreatment levels of SCC-Ag (>3.5 ng/mL) indicated a poor response to NACT and a higher risk of lymph node metastases. Elevated posttreatment levels of SCC-Ag were correlated with poor DFS and OS.

## Introduction

Cervical cancer is a significant cause of death in women worldwide, and approximately 250,000 patients with cervical cancer die every year.[1] In recent years, the use of neoadjuvant chemotherapy (NACT) has received increasing attention and has been used as an effective treatment in patients with cervical cancer. Compared with radiotherapy, NACT is more likely to improve the quality of life and psychosexual dysfunction.[2] In addition, neoadjuvant chemotherapy can shrink tumors prior to surgery, eliminate subclinical lesions and reduce the risk of lymph node metastases (LNM); therefore, it is used in Asia, Italy, South America and in many other countries.[3] NACT combined with radical surgery was also used for several years in China in patients with FIGO stage IB1-IIB cervical cancer.[2]

The majority (>85%) of cervical cancers are of the squamous cell type.[4] Squamous cell carcinoma antigen (SCC-Ag), a subfraction of tumor antigen TA-4, has been identified as a predictive and prognostic factor for squamous cell cervical carcinoma.[5] Pretreatment SCC-Ag levels are related to the FIGO stage, as it is elevated in approximately 24–53% and 75–90% of patients with Stage IB or IIA and Stage IIB and higher, respectively.[6–11] The pretreatment SCC-Ag level has been shown in earlier reports to be an independent indicator of chemotherapeutic response in patients with cervical cancer.[12] Scambia et al reported that the pretreatment level of SCC-Ag in non-responders (those with stable disease and progressive disease) to NACT is significantly higher than that in responders (complete response and partial response).[12] In addition, an elevated pretreatment level of SCC-Ag was found to be related to pelvic lymph node metastases.[7, 9, 10, 12–18] Different cutoff values were used to predict the status of lymph node metastases, and a higher cutoff value for the pretreatment level of SCC-Ag may be associated with a higher rate of metastases to the lymph node.[12] In several reports, an elevated pretreatment level of SCC-Ag was demonstrated to be an independent risk factor for poor survival.[7, 10, 12, 13]

However, the SCC-Ag level might be altered by treatment with NACT. Scambia et al reported that the variation in SCC-Ag levels and the response to neoadjuvant chemotherapy were significantly correlated. However, the impact of the changes in the serum SCC-Ag level after different NACT cycles on the chemotherapeutic response has never been extensively investigated. Additionally, the posttreatment (after NACT) SCC-Ag level, which is altered by NACT, has rarely been discussed. The significant correlation between the posttreatment SCC-Ag level and the response to NACT was only mentioned by Scambia.[12] The predictive value of the posttreatment levels of SCC-Ag as well as the relationship between the pretreatment levels of SCC-Ag and the posttreatment levels of SCC-Ag need to be evaluated.

We investigated the levels of SCC-Ag in the serum of all patients with cervical cancer who were treated with neoadjuvant chemotherapy followed by radical hysterectomy in order to clarify the role of the SCC-Ag level in the management of cervical cancer, especially the posttreatment SCC-Ag level.

## Patients and Methods

### Patients

A total of 286 patients with cervical cancer who were treated at Tongji Hospital in Wuhan, China from August 2008 to November 2012 were retrospectively enrolled in this study. Inclusion criteria were as follows: 1) patients with stage IB1–IIIB according to the Federation of Gynecology and Obstetrics (FIGO); 2) patients whose pathologic examinations showed squamous cell carcinoma of the cervix; 3) patients who did not receive prior hysterectomy, pelvic radiotherapy or concurrent chemoradiotherapy; 4) patients who were treated with neoadjuvant chemotherapy followed by radical hysterectomy; 5) patients with SCC-Ag levels measured before, during and after NACT. The patient enrollment flow was shown in **S1 Fig**. This study was approved by the Ethics Committee of Tongji Hospital of Tongji Medical College, Huazhong University of Science and Technology, PR China. All participants provided their written consent to participate in this study.

### Neoadjuvant chemotherapy regimens

All enrolled patients received 1–2 cycles of NACT every 28 days according to a predefined regimen. Generally, patients with early stage (IB1-IIA) cervical cancer received 1 cycle of NACT, while patients with advanced stage (IIB-IIIB) cancer received 2 cycles of NACT. However, the cycles of NACT that the patients received were based on the physician’s judgment. The regimens for NACT consisted of paclitaxel and cisplatin (TP) or irinotecan and cisplatin (CP). The response to neoadjuvant chemotherapy was evaluated according to the World Health Organization (WHO) criteria: CR, complete response; PR, partial response; SD, stable disease; PD, progressive disease.[2]

### Measurement of SCC-Ag levels

Serum samples were collected on day 1 of each cycle of NACT and before surgery. The SCC-Ag level was measured using an immunoradiometric assay kit (Imx, Abbott Diagnostics, Abbott Park, IL, USA). The normal upper limit for SCC-Ag was 1.5 ng/mL.

### Statistical analyses

Either Pearson’s chi-square test or Fisher’s exact test was used to assess the different patterns of SCC-Ag levels according to the clinicopathologic characteristics or the response to NACT. A receiver operating characteristic (ROC) curve was used to determine the best cutoff point for the levels of SCC-Ag in the serum in order to predict the response to chemotherapy. The risk factors for NACT and lymph node metastases were found by logistic regression analysis. The overall survival (OS) and progression-free survival (DFS) curves were calculated by using the Kaplan-Meier method with the log-rank test. Variables with *P* values <0.2 in the univariate analysis were selected for the Cox proportional-hazards model. All statistical analyses were performed using SPSS 13.0 software package (SPSS, Inc., Chicago, IL). *P* values <0.05 were considered significant in all statistical analyses.

## Results

A total of 286 patients with squamous cell carcinoma of the cervix were enrolled in this study. The number of patients with early stage (IB1-IIA) and advanced stage (IIB-IIIB) cancer was 135 and 151, respectively. The median age of these patients was 45 years (range: 22–72). The median follow-up time was 25.8 months, with a range of 6–81 months. A total of 153 patients received one cycle of NACT, and the other 133 patients received two cycles of NACT. All of the patients underwent radical surgery after NACT. The median value of the pretreatment and posttreatment levels of SCC-Ag was 3.0 ng/mL (range: 0.1–70.0) and 1.1 ng/mL (range: 0.0–179.0), respectively.

### Correlation between SCC-Ag levels and various clinicopathologic parameters

**Table 1** shows the distribution of SCC-Ag levels before and after NACT with respect to the clinicopathologic parameters in patients with squamous cell carcinoma of the cervix. Elevated pretreatment levels of SCC-Ag (>3.5 ng/mL) in the serum were associated with larger tumors, deep stromal infiltration (>1/3), and lymph node metastases (*P* = 0.027, *P* = 0.041, and *P*<0.001, respectively). Elevated posttreatment levels of SCC-Ag in the serum were associated with deep stromal infiltration and lymph node metastases (*P* = 0.006, and *P*<0.001, respectively). The level of SCC-Ag in the serum was correlated with the cycle of NACT before NACT, but not after NACT (*P* = 0.008, and *P* = 0.463, respectively).

**Table 1:** Pre- and posttreatment squamous cell carcinoma antigen serum levels according to different clinicopathologic variables.

	Pretreament SCC-Ag level (ng/mL)			Posttreament SCC-Ag level (ng/mL)		
Variable	≤3.5	>3.5	*P*	≤3.5	>3.5	*P*
Mean, range	3.0 (0.1–70.0)			1.1 (0.0–179.0)		
Age(years)						
≤35	9 (7.3%)	8 (8.3%)	0.780	15 (7.2%)	3 (9.7%)	0.633
>35	114 (92.7%)	88 (91.7%)		192 (92.8%)	28 (90.3%)	
FIGO stage						
IB1-IIA	58 (47.2%)	44 (45.8%)	0.846	102 (49.3%)	15 (46.9%)	0.800
IIB-IIIB	65 (52.8%)	52 (54.2%)		105 (50.7%)	17 (53.1%)	
Tumor size						
<4	37 (30.8%)	17 (17.7%)	0.027	66 (33.2%)	8 (25.0%)	0.358
≥4	83 (69.2%)	79 (82.3%)		133 (66.8%)	24 (75.0%)	
Grade						
Good or moderate	72 (72.7%)	56 (70.0%)	0.688	121 (70.8%)	18 (72.0%)	0.899
Poor	27 (27.3%)	24 (30.0%)		50 (29.2%)	7 (28.0%)	
NACT cycle						
1	74 (59.7%)	40 (37.7%)	0.008	105 (50.7%)	14 (43.8%)	0.463
2	50 (40.3%)	56 (52.8%)		102 (49.3%)	18 (56.3%)	
Parametrial invasion						
Negative	122 (98.4%)	96 (100.0%)	0.506^a^	204 (98.6%)	32 (100.0%)	1.000^a^
Positive	2 (1.6%)	0 (0.0%)		3 (1.4%)	0 (0.0%)	
Lymphovascular invasion						
Negative	123 (99.2%)	94 (97.9%)	0.582^a^	202 (97.6%)	32 (100.0%)	1.000^a^
Positive	1 (0.8%)	2 (2.1%)		5 (2.4%)	0 (0.0%)	
Lymph node metastases						
Negative	116 (93.5%)	62 (64.6%)	<0.001	178 (86.0%)	19 (59.4%)	<0.001
Positive	8 (6.5%)	34 (35.4%)		29 (14.0%)	13 (40.6%)	
>1/3 stromal infiltration						
No	79 (63.7%)	48 (50.0%)	0.041	125 (60.4%)	11 (34.4%)	0.006
Yes	45 (36.3%)	48 (50.0%)		82 (39.6%)	21 (65.6%)	

SCC-Ag, squamous cell carcinoma antigen; FIGO, International Federation of Gynecology and Obstetrics; NACT, neoadjuvant chemotherapy.

^a^ *P* value calculated using fisher's exact test

### The relationship between NACT and serum SCC-Ag levels

Overall, the proportion of patients with elevated serum levels of SCC-Ag (>3.5 ng/mL) was 43.8% (96/219) before the original treatment and only 13.0% (31/238) after NACT (*P*<0.001) (**Table 2**). The effect was still significant after stratification by NACT cycle (*P*<0.001 for both 1 and 2 cycles).

**Table 2:** Impact of neoadjuvant chemotherapy on SCC-Ag levels.

	SCC-Ag level		
	≤3.5 (%)	>3.5 (%)	*P*
Overall			
Pretreatment	123(56.2)	96(43.8)	<0.001
posttreatment	207(87.0)	31(13.0)	
NACT cycle			
1 cycle			
Pretreatment	74(64.9)	40(35.1)	<0.001
posttreatment	105(88.2)	14(11.8)	
2 cycles			
Pretreatment	50(47.2)	56(52.8)	<0.001
posttreatment	102(85.0)	18(15.0)	

SCC-Ag, squamous cell carcinoma antigen; NACT, neoadjuvant chemotherapy.

### The Relationship between the response to NACT and the different patterns of serum SCC-Ag levels

The overall clinical response rate of the patients to NACT was 76.6% (210/274). As shown in **S1 Table**, both pretreatment and posttreatment levels of SCC-Ag in the serum (>3.5 ng/mL) were related to the clinical response to neoadjuvant chemotherapy (*P* = 0.010, and *P*<0.001, respectively). After conducting a stratified analysis, we found that the posttreatment SCC-Ag levels were related to the response to NACT in patients who received both 1 and 2 cycles of NACT (*P* = 0.006, and *P*<0.001, respectively). The pretreatment level of SCC-Ag was significantly related to the clinical response to NACT in patients who received 1 cycle of NACT (*P* = 0.047), but not 2 cycles of NACT (*P* = 0.095). In the multivariate analysis, the pretreatment level of SCC-Ag (>3.5 ng/mL) was demonstrated to be an independent risk factor for poor clinical response to neoadjuvant chemotherapy (*P* = 0.025) (**Table 3**).

**Table 3:** Univariate and multivariate analysis for clinical response of neoadjuvant chemotherapy.

					Univariate	Multivariate
	CR	PR	CR+PR	SD+PD	*P*	*P*
Age(years)						
≤35	4	13	17	4	0.792^a^	
>35	29	164	193	59		
FIGO stage						
IA-IIA	17	80	97	34	0.347	
IIB-III	16	96	112	30		
Tumor size						
<4	20	45	65	15	0.184	
≥4	11	126	137	49		
Grade						
Good or moderate	14	107	121	36	0.292	
Poor	1	44	45	19		
NACT cycle						
1	19	91	110	31	0.581	
2	14	86	100	33		
Pretreatment SCC-Ag level						
≤3.5	17	81	98	22	0.010	0.025
>3.5	5	56	61	31		

CR, complete response; PR, partial response; SD, stable disease; PD, progressive disease; FIGO, International Federation of Gynecology and Obstetrics; NACT, neoadjuvant chemotherapy; SCC-Ag, squamous cell carcinoma antigen.

^a^ *P* value calculated using fisher's exact test.

### Survival analysis

The follow-up rate was 73.1% (209/286). In the univariate analysis, tumor size, lymphovascular invasion, and posttreatment level of SCC-Ag were significant predictors of disease-free survival. FIGO stage, number of cycles of NACT, posttreatment level of SCC-Ag, and lymph node metastases all had prognostic significance for overall survival (**Table 4**). The variables with a *P* value <0.2 were used to perform the multivariate analysis. We found that the posttreatment SCC-Ag level (>3.5 ng/mL) was an independent risk factor for both disease-free survival and overall survival (*P* = 0.012, and *P* = 0.001, respectively). While lymphovascular invasion was found to be an independent risk factor for DFS, advanced FIGO stage (IIB-IIIB) and lymph node metastases were determined to be independent risk factors for OS.

**Table 4:** Univariate and multivariate analysis for disease-free survival and overall survival.

		3-DFS			3-OS		
	N	Univariate	*P*	Multivariate	Univariate	*P*	Multivariate
Age(years)							
≤35	16	87.5%	0.969		87.5%	0.857	
>35	193	92.3%			85.1%		
FIGO stage							
IA-IIA	96	90.8%	0.586		94.5%	0.017	0.036
IIB-III	112	92.5%			77.8%		
Tumor size							
<4	65	100.0%	0.030		87.2%	0.961	
≥4	137	87.9%			83.4%		
Grade							
Good or moderate	121	90.4%	0.458		84.6%	0.455	
Poor	43	91.3%			80.4%		
NACT cycles							
1	111	95.3%	0.098		91.3%	0.019	
2	98	87.0%			76.8%		
Pretreatment SCC-Ag level							
≤3.5	61	93.1%	0.221		85.9%	0.178	
>3.5	101	87.8%			81.3%		
Posttreatment SCC-Ag level							
≤3.5	134	94.9%	<0.001	0.012	89.7%	<0.001	0.001
>3.5	42	76.0%			60.1%		
Parametrial invasion							
Negative	207	91.7%	0.683		85.0%	0.574	
Positive	2	100.0%			100.0%		
Lymphovascular invasion							
Negative	205	92.1%	0.050	0.006	85.4%	0.178	
Positive	4	75.0%			75.0%		
Lymph node metastases							
Negative	171	93.7%	0.081		89.6%	<0.001	0.019
Positive	38	82.2%			63.4%		
>1/3 stromal infiltration							
Negative	128	93.0%	0.549		87.4%	0.120	
Positive	81	90.1%			82.3%		

DFS, disease-free survival; OS, overall survival; FIGO, International Federation of Gynecology and Obstetrics; NACT, neoadjuvant chemotherapy.

**Fig 1:**
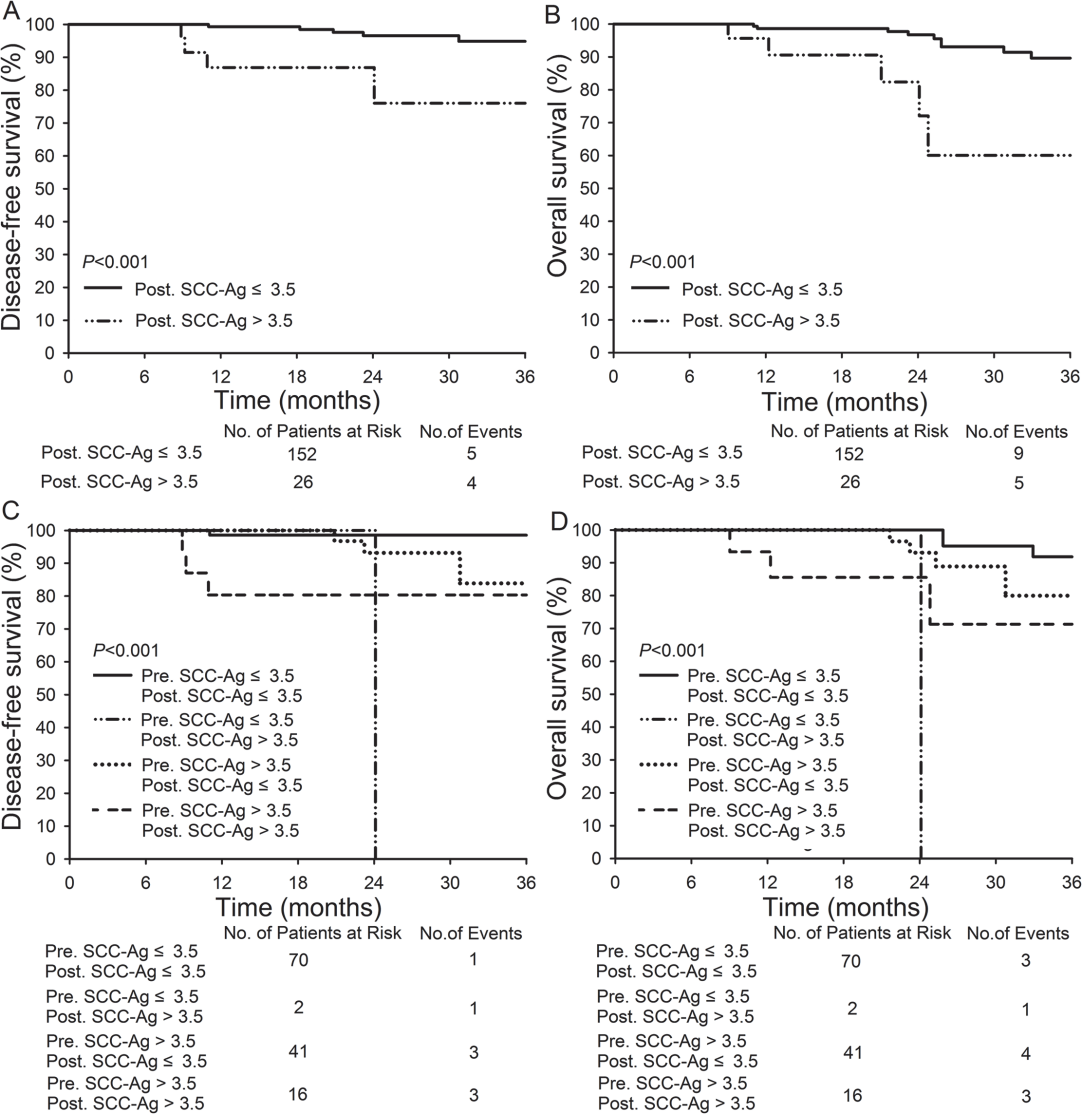
Disease-free survival and overall survival of patients in different SCC-Ag subgroups. (A-B): Comparison of DFS and OS between patients with posttreatment SCC-Ag level ≤3.5 ng/mL (n = 152) and >3.5 ng/mL (n = 26); (C-D): Comparison of DFS and OS in patients with pretreatment SCC-Ag ≤3.5 ng/mL and posttreatment SCC-Ag ≤3.5 ng/mL (n = 70), patients with pretreatment SCC-Ag ≤3.5 ng/mL and posttreatment SCC-Ag >3.5 ng/mL (n = 2), patients with pretreatment SCC-Ag >3.5 ng/mL and posttreatment SCC-Ag ≤ 3.5 ng/mL (n = 41), and patients with pretreatment SCC-Ag > 3.5 ng/mL and posttreatment SCC-Ag >3.5 ng/mL four subgroups (n = 16).

The difference in the 3-year DFS and 3-year OS for patients with posttreatment SCC-Ag levels ≤3.5 ng/mL and >3.5 ng/mL was significant: 94.9% and 89.7% versus 76.0% and 60.1%, respectively (*P*<0.001 for both) (**Fig 1A and 1B**). With respect to the response to NACT, no significant difference was observed between responders and non-responders in the 3-year DFS and 3-year OS (**S2 Table**). We divided the patients into four subgroups according to pre- and posttreatment levels of SCC-Ag. It showed that the patients with both pre- and posttreatment SCC-Ag levels ≤3.5 ng/mL demonstrated the best 3-year DFS (98.6%, *P*<0.001) and 3-year OS (91.8%, *P*<0.001) compared with the other three subgroups (**Fig 1C and 1D**). When we compared pathological features and clinical response to NACT among the four subgroups, patients with both pre- and posttreatment SCC-Ag levels >3.5 ng/mL showed lower clinical response to NACT (31.8%), higher poor grade rate (31.3%), more tumor size ≥4 cm (86.4%), higher rate of Lymph node metastases (45.5%), and more >1/3 stromal infiltration positive (63.6%). The number of patients (n = 3) with pretreatment SCC-Ag ≤3.5 ng/mL and posttreatment SCC-Ag >3.5 ng/mL was too small to be compared (**S3 Table**).

### The predictive significance of SCC-Ag level in terms of pathologic factors

As described earlier, higher pretreatment and posttreatment levels of SCC-Ag (>3.5 ng/mL) were associated with lymph node metastases and >1/3 stromal infiltration (**Table 1**). Therefore, we performed a multivariate analysis by using the logistic model to evaluate the relationship between SCC-Ag levels and the two pathologic factors. We found that only an elevated pretreatment level of SCC-Ag level (>3.5 ng/mL) was an independent risk factor for lymph node metastases (**Table 5**), while poor tumor grade was the only independent risk factor for deep stromal infiltration (>1/3). Posttreatment levels of SCC-Ag alone could not predict lymph node metastases and deep stromal infiltration.

**Table 5:** Univariate and multivariate analysis for lymph node metastases and deep stromal infiltration.

	Lymph node metastases			Univariate	Multivariate	>1/3 stromal infiltration			Univariate	Multivariate
Variable	Negative		Positive	*P*	*P*	No		Yes	*P*	*P*
Age(years)										
≤35	19		4	1.000^a^		12		11	0.514	
>35	215		47			155		107		
FIGO stage										
IB1-IIA	115		20	0.198		79		56	0.980	
IIB-IIIB	119		31			88		62		
Turmor size										
<4	68		14	0.77		54		28	0.085	
≥4	158		36			106		88		
Grade										
Good or moderate	133		26	0.414		92		67	0.009	0.027
Poor	53		14			26		41		
NACT cycles										
1	129		24	0.309		91		62	0.786	
2	106		27			77		56		
Pretreatment SCC-Ag level										
≤3.5	116		8	<0.001	<0.001	79		45	0.041	
>3.5	62		29			48		48		
Posttreatment SCC-Ag level										
≤3.5	178		29	<0.001		125		82	0.006	
>3.5	19		13			11		21		

FIGO, International Federation of Gynecology and Obstetrics; NACT, neoadjuvant chemotherapy; SCC-Ag, squamous cell carcinoma antigen.

^a^ *P* value calculated using fisher's exact test.

## Discussion

In many previous reports, elevated pretreatment levels of SCC-Ag were found to correlate with unfavorable clinicopathologic characteristics, such as advanced stage, larger tumor size, deep stromal infiltration, and lymph node metastasis.[7, 8, 19–21] We found comparable results in the current study (**Table 1**). The cutoff value of pretreatment levels of SCC-Ag varies among different studies. Massuger et al[22] and Takeshima et al[15] used cutoff values of 2.5 ng/mL and 4 ng/mL, respectively, of pretreatment levels of SCC-Ag in order to predict lymph node metastases. Lin et al[17] reported that approximately 65% of the patients with serum levels of SCC-Ag >8 ng/mL demonstrated lymph node metastases. In this study, the elevated pretreatment level of SCC-Ag (>3.5 ng/mL) was shown to be the only independent risk factor for lymph node metastasis, and this has also been reported in several other studies.[16, 18, 19] At the same time, we could not ignore the patients whose pretreatment SCC-Ag levels were <3.5 ng/mL, as they also demonstrated a low rate of lymph node metastases.

Neoadjuvant chemotherapy has been proven to be an effective treatment for patients with locally advanced cervical cancer. We found that a pretreatment level of SCC-Ag >3.5 ng/mL was the only independent risk factor for poor clinical response to NACT, as reported by Scambia et al.[12] Pretreatment SCC-Ag values served as indicators of the original levels of SCC-Ag, which were not affected by any treatment. However, the levels of SCC-Ag could be altered by NACT. From **Table 2**, we know that NACT decreased the amount of elevated SCC-Ag (from 52.8%-35.1% to 15.0%-11.8%). At the same time, we found that tumor size and the number of cycles of NACT were significantly correlated with pretreatment levels of SCC-Ag, but were not correlated with posttreatment levels of SCC-Ag. With respect to the size of the tumors of the responders to NACT, tumor size could be reduced by NACT, it was not significantly related to the posttreatment SCC-Ag level. With regards to the number of cycles of NACT, the data suggest that the change in the levels of SCC-Ag was due to the NACT but not the number of cycles that the patients received.

What information could the adjusted (i.e., posttreatment) SCC-Ag value provide? First, the SCC-Ag level was related to the clinical response to NACT, whether the patients received one or two cycles of NACT (**S1 Table**). Second, the SCC-Ag level was related to two pathologic factors, lymph node metastases (*P*<0.001) and >1/3 stromal infiltration (*P* = 0.006), but demonstrated no significant relationship with parametrial invasion and lymphovascular invasion. Although the pretreatment level of SCC-Ag was also related to lymph node metastases and >1/3 stromal infiltration in the univariate analysis, the posttreatment level of SCC-Ag showed a significant association with survival when a multivariate analysis was performed. A posttreatment level of SCC-Ag >3.5 ng/mL was shown to be an independent predictive factor for both disease-free survival and overall survival.

From the above analysis, neoadjuvant chemotherapy before radical surgery seems to be a selector which divided the patients into those with improved survival times and those with poor survival times by using the change of SCC-Ag. When the pre- and posttreatment SCC-Ag levels were combined, we found that the patients with pretreatment levels of SCC-Ag <3.5 ng/mL rarely demonstrated elevated (>3.5) posttreatment levels of SCC-Ag (only two patients in this study, one of whom died at 24 months). Patients with both pre- and posttreatment levels of SCC-Ag <3.5 ng/mL showed the best 3-year disease-free survival rate (98.6%) and overall survival rate (91.8%) compared with the other three subgroups (**Fig 1C and 1D**). Patients with SCC-Ag >3.5 ng/mL, specifically at the posttreatment time point, require more attention from their physicians. Though their response to NACT had no significant relationship with survival, NACT changed the status of their serum SCC-Ag levels, which was an excellent predictor of survival.

The different effects of pre- and posttreatment levels of SCC-Ag may inform us that the treatment with NACT decreased the tumor size, but did not result in a great improvement of the pathologic condition of the patients. Ting Hu et al[2] also reported that the pathologic factors between patients with or without NACT before radical surgery showed no significant difference, although NACT did improve the 5-year DFS and OS of the patients.

In conclusion, the pretreatment SCC-Ag level is an independent factor for pelvic lymph node metastasis and neoadjuvant chemotherapy response in patients with cervical cancer. An elevated posttreatment level of SCC-Ag level (>3.5 ng/mL) is an independent risk factor of both disease-free survival and overall survival. Patients with either pretreatment or posttreatment levels of SCC-Ag >3.5 ng/mL should be given more attention. Due to the limits inherent in retrospective studies, additional studies are needed to validate the importance of SCC-Ag and its prognostic value.

## Supporting Information

S1 Fig Patient enrollment in the present study. (TIF)

S1 Table Relationship between pre-, and posttreatment SCC-Ag levels and NACT response. (DOCX)

S2 Table Univariate survival analysis for clinical response to neoadjuvant chemotherapy. (DOCX)

S3 Table Compare of clinic-pathological factors among patients in subgroups divided by pre- and posttreatment levels of SCC-Ag. (DOCX)
