# Supplementary figures and images for: The Predictive Value of Serum Squamous Cell Carcinoma Antigen in Patients with Cervical Cancer Who Receive Neoadjuvant Chemotherapy followed by Radical Surgery: A Single-Institute Study
Source: PLoS One. 2015 Apr 10;10(4):e0122361. doi: 10.1371/journal.pone.0122361 (PMC4393273; doi:10.1371/journal.pone.0122361)

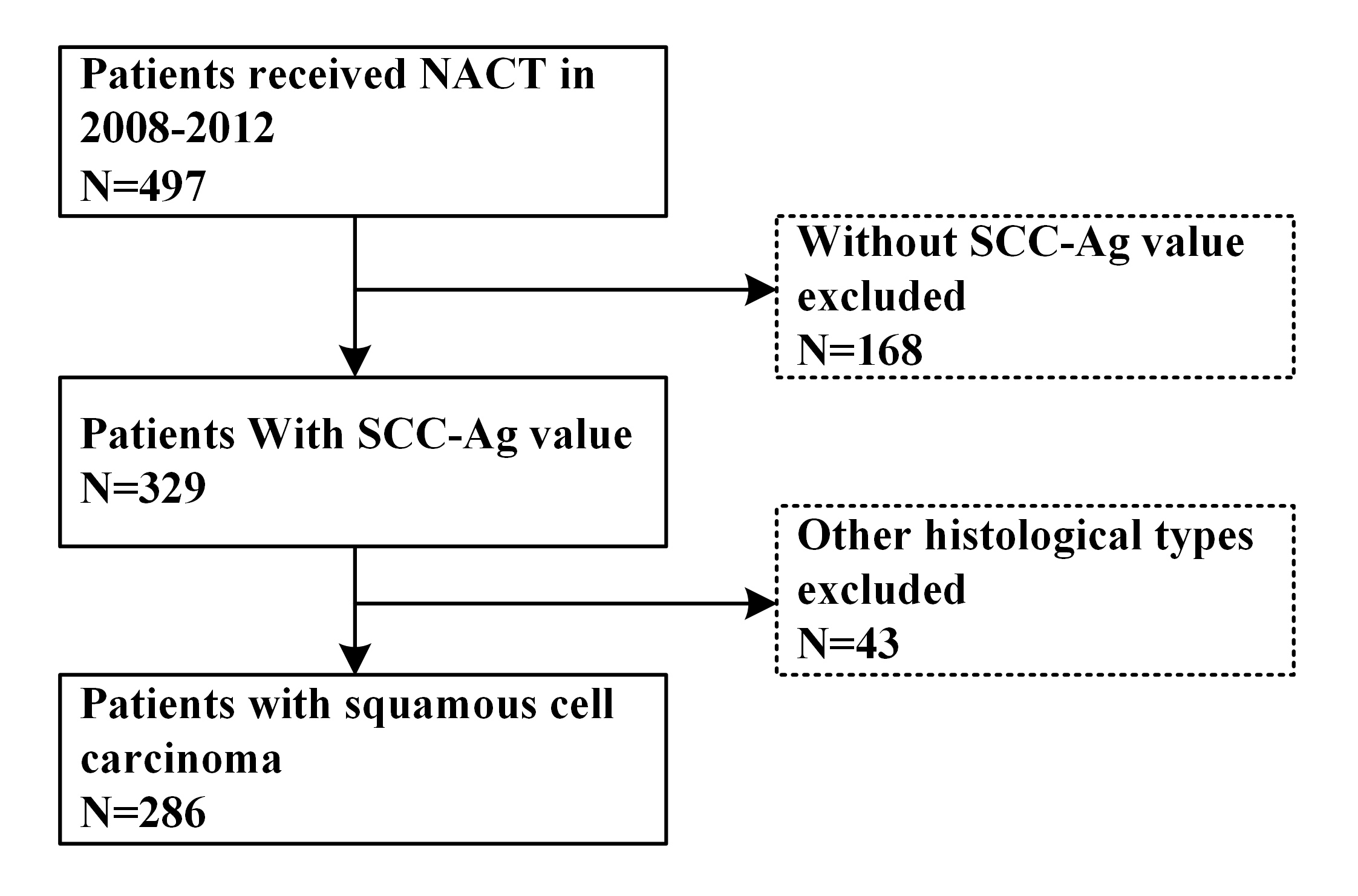

Supplement: S1 Fig — (TIF) [file pone.0122361.s001.tif]
