# Supplementary material for: The Predictive Value of Serum Squamous Cell Carcinoma Antigen in Patients with Cervical Cancer Who Receive Neoadjuvant Chemotherapy followed by Radical Surgery: A Single-Institute Study
Source: PLoS One. 2015 Apr 10;10(4):e0122361. doi: 10.1371/journal.pone.0122361 (PMC4393273; doi:10.1371/journal.pone.0122361)
Supplement: S1 Table — (DOCX) [file pone.0122361.s002.docx]

**S1_Table.** Relationship between pre-, and posttreatment SCC-Ag levels and NACT response.

|  | Pretreatment SCC-Ag (ng/mL) | |  |  | Posttreatment SCC-Ag (ng/mL) | |  |
| --- | --- | --- | --- | --- | --- | --- | --- |
| Clinical response | ≤3.5 | >3.5 | P |  | ≤3.5 | >3.5 | *P* |
| Overall |  |  |  |  |  |  |  |
| CR | 17 | 5 |  |  | 26 | 1 |  |
| PR | 81 | 56 |  |  | 119 | 31 |  |
| CR+PR | 98 | 61 | 0.010 |  | 165 | 12 | <0.001 |
| SD+PD | 22 | 31 |  |  | 36 | 20 |  |
| NACT cycle |  |  |  |  |  |  |  |
| 1 cycle |  |  |  |  |  |  |  |
| CR+PR | 57 | 23 | 0.047 |  | 79 | 6 | 0.006^a^ |
| SD+PD | 13 | 13 |  |  | 20 | 8 |  |
| 2 cycles |  |  |  |  |  |  |  |
| CR+PR | 41 | 38 | 0.095 |  | 86 | 6 | <0.001 |
| SD+PD | 9 | 18 |  |  | 16 | 12 |  |

a. P value calculated using fisher's exact test.
