# Supplementary material for: The Predictive Value of Serum Squamous Cell Carcinoma Antigen in Patients with Cervical Cancer Who Receive Neoadjuvant Chemotherapy followed by Radical Surgery: A Single-Institute Study
Source: PLoS One. 2015 Apr 10;10(4):e0122361. doi: 10.1371/journal.pone.0122361 (PMC4393273; doi:10.1371/journal.pone.0122361)
Supplement: S2 Table — (DOCX) [file pone.0122361.s003.docx]

**S2_Table.** Univariate survival analysis for clinical response to neoadjuvant chemotherapy.

|  |  | 3-DFS | |  | 3-OS | | |
| --- | --- | --- | --- | --- | --- | --- | --- |
|  | N | Univariate | *P* |  | Univariate | *P* |  |
| NACT |  |  |  |  |  |  | |
| CR+PR | 157 | 91.9% | 0.318 |  | 86.9% | 0.154 | |
| SD+PD | 45 | 89.4% |  |  | 79.4% |  | |
| Post-treatment SCCA level |  |  |  |  |  |  | |
| ≤3.5 |  |  |  |  |  |  | |
| CR+PR | 124 | 93.6% | 0.312 |  | 91.2% | 0.345 | |
| SD+PD | 24 | 100.0% |  |  | 87.2% |  | |
| >3.5 |  |  |  |  |  |  | |
| CR+PR | 9 | 100.0% | 0.142 |  | 88.9% | 0.474 | |
| SD+PD | 15 | 66.2% |  |  | 48.7% |  | |
