# Supplementary material for: The Predictive Value of Serum Squamous Cell Carcinoma Antigen in Patients with Cervical Cancer Who Receive Neoadjuvant Chemotherapy followed by Radical Surgery: A Single-Institute Study
Source: PLoS One. 2015 Apr 10;10(4):e0122361. doi: 10.1371/journal.pone.0122361 (PMC4393273; doi:10.1371/journal.pone.0122361)
Supplement: S3 Table — (DOCX) [file pone.0122361.s004.docx]

**S3_Table.** Compare of clinic-pathological factors among patients in subgroups divided by pre- and posttreatment levels of SCC-Ag.

|  |  | pretreatment SCC-Ag ≤3.5 | |  | pretreatment SCC-Ag >3.5 | |  |
| --- | --- | --- | --- | --- | --- | --- | --- |
|  |  | posttreatment SCC-Ag | |  | posttreatment SCC-Ag | |  |
|  |  | ≤3.5 | >3.5 |  | ≤3.5 | >3.5 | *P* value |
| FIGO stage |  |  |  |  |  |  |  |
| IB1-IIA |  | 46 (51.1%) | 1 (33.3%) |  | 25 (43.1%) | 12 (54.5%) | 0.678 |
| IIB-IIIB |  | 44 (48.9%) | 2 (66.7%) |  | 33 (56.9%) | 10 (45.5%) |  |
| Clinical response |  |  |  |  |  |  |  |
| CR+PR |  | 71 (79.8%) | 1 (33.3%) |  | 47 (82.5%) | 7 (31.8%) | <0.001 |
| SD+PD |  | 18 (20.2%) | 2 (66.7%) |  | 10 (17.5%) | 15 (68.2%) |  |
| Grade |  |  |  |  |  |  |  |
| Good or moderate |  | 57 (75.0%) | 2 (66.7%) |  | 38 (70.4%) | 11 (68.8%) | 0.913 |
| Poor |  | 19 (25.0%) | 1 (33.3%) |  | 16 (29.6%) | 5 (31.3%) |  |
| Tumor size |  |  |  |  |  |  |  |
| <4 cm |  | 29 (33.0%) | 2 (66.7%) |  | 12 (20.7%) | 3 (13.6%) | 0.067 |
| ≥4 cm |  | 59 (67.0%) | 1 (33.3%) |  | 46 (79.3%) | 19 (86.4%) |  |
| Parametrial invasion |  |  |  |  |  |  |  |
| Negative |  | 88 (97.8%) | 3 (100.0%) |  | 58 (100.0%) | 22 (100.0%) | 0.601 |
| Positive |  | 2 (2.2%) | 0 (0.0%) |  | 0 (0.0%) | 0 (0.0%) |  |
| Lymphovascular invasion |  |  |  |  |  |  |  |
| Negative |  | 89 (98.9%) | 3 (100.0%) |  | 57 (98.3%) | 22 (100.0%) | 0.928 |
| Positive |  | 1 (1.1%) | 0 (0.0%) |  | 1 (1.7%) | 0 (0.0%) |  |
| Lymph node metastases |  |  |  |  |  |  |  |
| Negative |  | 85 (94.4%) | 3 (100.0%) |  | 40 (69.0%) | 12 (54.5%) | <0.001 |
| Positive |  | 5 (5.6%) | 0 (0.0%) |  | 18 (31.0%) | 10 (45.5%) |  |
| >1/3 stromal infiltration |  |  |  |  |  |  |  |
| No |  | 57 (63.3%) | 2 (66.7%) |  | 28 (48.3%) | 8 (36.4%) | 0.077 |
| Yes |  | 33 (36.7%) | 1 (33.3%) |  | 30 (51.7%) | 14 (63.6%) |  |

SCC-Ag, squamous cell carcinoma antigen; FIGO, International Federation of Gynecology and Obstetrics; CR, complete response; PR, partial response; SD, stable disease; PD, progressive disease;
